# The quest for lower alcoholic wines

**DOI:** 10.1111/1751-7915.12594

**Published:** 2017-01-29

**Authors:** Antonio Caballero, Ana Segura

**Affiliations:** ^1^ BacmineParque Científico de Madrid28760Tres CantosSpain; ^2^ Estación Experimental del ZaidínCSICProfesor Albareda 118008GranadaSpain

## Abstract

Wine industry is engaged in finding technological ways to decrease alcohol concentration in wines without spoiling their organoleptic properties. Such challenge requires, among other strategies, modification of the yeast strains carrying out the fermentation. In this issue of Microb. Biotechnol., Goold and colleagues have reviewed one of the most straightforward yeast modification, altering its metabolism to produce glycerol instead of alcohol.

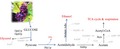

According to the International Wine Organization (OIV), the estimated global wine production in 2015 reached 274 millions of hectolitres (mhl); 239 mhl was consumed during the same year and, as an informative number, 43% of the total wine was consumed in a different country from where it was produced. In agreement with this foreign consume, wine trading increased a 10% in value in 2015, reaching 28 billion EUR. This means a shift in the trend of wine consumption, from localized in the country where it is produced, to a more globalized market. Principally, what this means to wine producers is that they have to adapt their production to the competitiveness of the worldwide market, and they are required to develop a product able to satisfy the taste and demands of more diverse consumers. Accordingly, the tendency of the consumer is imposing rich‐flavoured, well‐balanced and full‐bodied wines. These types of wines are usually achieved through late harvesting to get completely ripened grapes. Such ripened fruits are richer in sugar content, phenolic maturity and aromatic complexity. However, these sorts of properties are usually associated with wines presenting higher ethanol content. Indeed, ethanol concentration has increased an average of a 2% (v/v) in the last thirty years. Moreover, experts foresee even more increased ethanol content due to the effects of climate change, as this will provoke grape to mature faster, becoming more sugared (Jones *et al*., 2005).

This lately evolution in the wine composition is confronted by serious efforts to reduce the presence of ethanol in wine. Both industry and public pursue this interest for many reasons. First, for the wine industry, ethanol is a component that may affect wine flavour perception, hampering wine quality, and, additionally, such amounts of ethanol could poison the must, and might provoke sluggish or even stuck fermentations. Furthermore, ethanol taxation in some countries affects negatively wine competitiveness. But more importantly, alcohol consumption is also a major concerning issue for public health. For those reasons, many have embarked in order to obtain research and technology towards finding ways to reduce ethanol concentration without affecting organoleptic properties accompanying prestigious, high‐quality wines.

As a matter of fact, wine composition, texture, colour, aroma and flavour are affected by every single step during wine production. A whole succession of factors that should be treated as variables, that include the soil composition, temperature and weather conditions, size of the vine and the leaves, humidity, grape maturation, plague control, harvesting conditions, among other environmental and human factors, cluster to influence wine characteristics. But furthermore, fermentation is necessarily affected by the complexity of the existing microbiota in the starting must brought together with the berries, and finally by the chosen *Saccharomyces cerevisiae* strain or strains that perform the final fermentation. Under this combination of parameters, altering a single characteristic of the wine, in this case the ethanol content, and do it so without affecting the rest of the properties is the enormous and complex challenge that troubles equally scientist, engineers and experts.

Beyond many elaborated strategies focus on cultivation and selection of vine plants, or how to perform the harvesting (such as less sugar accumulating vines, adjusting the harvesting time to reduce glucose accumulation in less ripen grapes), one of the major strategies rationalized to tackle alcohol synthesis has focus in the principal culprit of ethanol production, the yeast *S. cerevisiae* and its formidable fermentative metabolism. A broad catalog of the scientific literature concerning this topic is summarized in this issue of *Microbial Biotechnology*. The review of Goold *et al*. (2016) have put its emphasis on the alternative genetic and molecular approaches to modify yeast metabolism, and more specifically, the attempts to redirect the carbon flux from ethanol to glycerol (see Fig. 1 for a graphic view of glucose metabolism in *S. cerevisiae*). Several methods are discussed in this review, explaining how such detour during ethanol fermentation might be achieved. One of the methods explored consists in breeding different *S. cerevisiae* strains to select less ethanol producer yeast. Indeed, this breed could involve different *Saccharomyces* species, where wine industrial workhorse strains could be mixed with known less alcoholic species. Indeed, such hybrid strains have been described with reduced efficiency regarding alcohol yields, and preserving some of the wine organoleptic properties after fermentation. Additionally, the yeast could be forced to evolve and adapt to conditions where glycerol synthesis is more favoured, for example conditioning the yeast to higher osmotic pressures or even higher sulphite concentrations. Nevertheless, these tactics are based on serendipitous events, requiring of multiple strain crossings to get the required metabolism, but obviating other changes that might be carried over. Hence, apart of time‐consuming, the behaviour of these strains is difficult to control and predict in changing environments occurring inside a winery.

**Figure 1:**
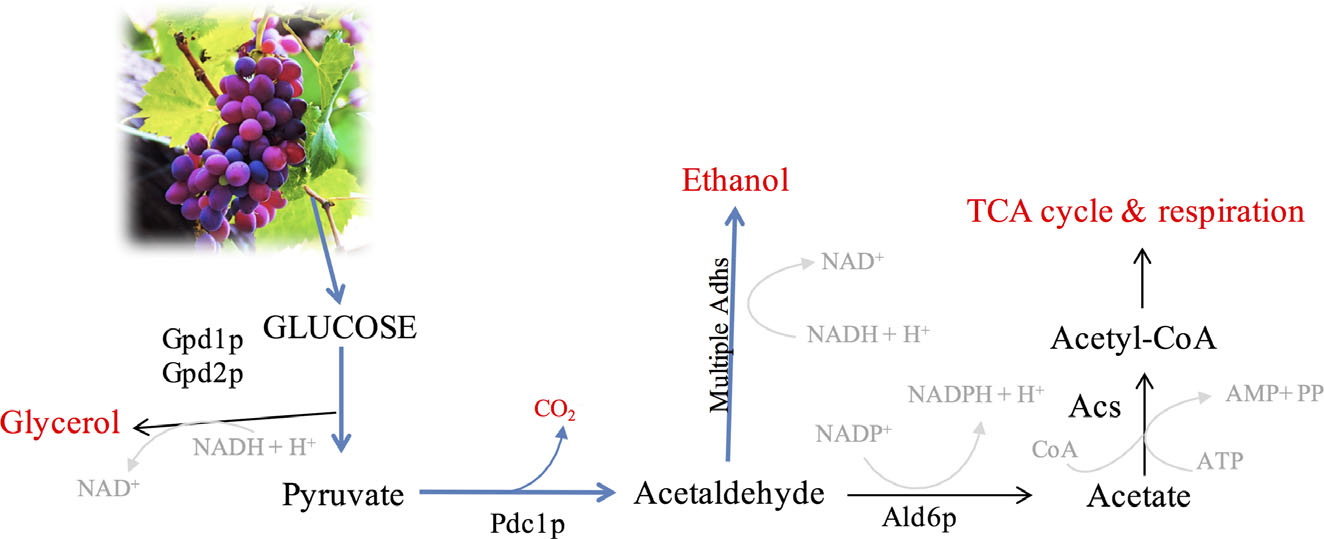
This figure depicts the major pathways for glucose consumption in *S. cereviseae*. In the presence of pyruvate (derived from the glycolysis), the favoured pathway (blue arrows) is the fermentation and production of ethanol. The synthesis of acetyl‐coA and the tricarboxylic acid cycle (TCA) are inhibited by catabolic repression due to the Crabtree effect. Eventually, and depending on the fermentation conditions and the available reducing power, products such as acetaldehyde, acetate and the derived product acetoin are accumulated at different proportions, influencing the final wine quality. On the other hand, glycerol is an osmotic protector. Gpd1p/Gpd2p use glyceraldehyde‐3‐phosphate derived from glycolysis, and its production is regulated according to cell demands.

Therefore, the direct and guided manipulation of the genome is probably the most efficient procedure, but the use of genetic modified yeast is nowadays beyond any winery current agenda, as unpopularity of the genetic modified organism (GMO) among consumers is even stronger than the legal restrictions associated with their use, especially in wine business, with strong roots in traditions. In this regard, the recent discovery and the rapid acceptance of DNA‐editing technologies based on CRIPSR/cas9 is probably the revolutionary solution that the biotechnology required to step forward, as genes could be easily altered without any incorporation of alien genetic material. Nevertheless, as summarized by Goold and colleagues, there are multiple different genetic modifications that have been assayed in many laboratories, to modify carbon fluxes, reduce ethanol content and enhance glycerol and/or CO_2_ production. In fact, significant reduced ethanol production has been achieved by overexpressing the genes encoding for the glycerol phosphate dehydrogenase (*GPD1* & *GPD2*) although these changes resulted in the accumulation of unwanted metabolites due to unbalanced redox metabolism. Unfortunately, those metabolites affect negatively the wine quality, and more genetic modifications are required to avoid this sort of secondary metabolism (Ehsani *et al*., 2009).

Solving these metabolic puzzles is not a straightforward duty when it comes to deriving fluxes of the central metabolism of the cell, especially if the protagonist is *S. cerevisiae,* whose robust metabolic machinery has been selected during centuries and forced to adapt with the only mission of fermenting and producing ethanol. In fact, most of the *S. cerevisiae* strains possess several *ADH* genes codifying for alcohol dehydrogenases (although the number might vary depending on the strain background), and it has been shown that mutating a single gene have no effect in ethanol production whatsoever. Due to compensatory mechanisms, no significant ethanol reduction is detected unless a combination of several *ADH* genes is knocked down at the same time (Ida *et al*., 2012; Hirasawa *et al*., 2014). This indicates that in order to achieve a desired amount of ethanol production, those compensatory genetic mechanisms have to be very well analysed and predicted. Alternatively, other modifications less discussed by Goold and colleagues and with relative success to reduce ethanol production have tried to counteract the Crabtree effect (different metabolism than alcoholic fermentation, such as respiration, is inhibited while glucose is available) through regulatory changes that stimulate respiration instead of fermentation (Van Maris *et al*., 2001; Raab *et al*., 2011). This has been partially achieved overexpressing the transcriptional factor Hap4p (regulator of the carbon source‐dependent respiratory activation) and Sak1p (activator of *SNF1,* a major regulator of glucose catabolic repression), but to our knowledge, such changes have not been tested under the wine production perspective (Raab *et al*., 2011). It might be interesting to test whether a combinatory of modifications to enhance respiration and glycerol synthesis together with a reduced amount of *ADHs* might generate a better metabolic panorama to reduce ethanol without major undesired side‐product accumulation.

A different perspective explored by Goold and colleagues is to carry out fermentations based on mixed population of species existing in the must would play a major role in fermentations, as they do not produce as much ethanol as *S. cerevisiae*. The problem argued Goold and colleagues is the difficulty of keeping the variable microbial ecosystem along fermentations, as this is influenced by many external factors. For example, the chosen phytosanitary protection and the amount of sulphites added have a dramatic impact on these mixed populations, which are critical to produce some of the wine characteristics (Grangeteau *et al*., 2016). Nevertheless, other authors favour the idea of mixed fermentations as a solution to decrease ethanol content (Ciani *et al*., 2016). According to their suggestions, if glycerol is the final desired product used as ethanol alternative during fermentation, in order to decrease a 2–3% alcohol content, glycerol have to rise up to five times more concentration than in the existing wines. In their perspective, controlled mixed fermentations (rather than ones combined with existing undefined microbiota) could be designed, combining *S. cerevisiae* with other species such as *Starmerella bombicola* or *Lachancea themotolerans* that reduce glucose content through respiration, while *S. cerevisiae* ensures fully fermentation and maintained major wine properties. In fact, rather than a negative impact in the fermentation, it probed to be somehow beneficial in order to reduce side‐product accumulation, as one organism is able to degrade what the other produces (such as acetoin or acetaldehyde). In fact, traditionally, wine production was based on spontaneous fermentations where multiple species collaborated together with *S. cerevisiae* to the final outcome. Although these fermentations are intrinsically risky, when it occurred successfully, those wines were acknowledged as better in texture and flavour profile (Jolly *et al*., 2014). In any case, such technology could be either fine‐tuned in wineries to produce rich metabolites that would improve organoleptic properties and reduce the accumulation of side unwanted products (readers are kindly directed to Jolly *et al*., 2014; Varela, 2016 for extensive updated reviews in non‐*Saccharomyces* fermenting yeast in the brewing industry). Whether this mixed and multiphased sequential fermentation occur between different species or combined *S. cerevisiae* strains either selected or modified *á la carte* for specific purposes is an interesting topic yet to investigate.

Conclusively, as a *gourmet* industry, flavour is the major selling point in the wine business. As a consequence, to success with the reduction of ethanol content without affecting the delicate equilibrium, complexity and combination of factors that rules the organoleptic wine properties, this will probably require more than just the modification of single parameter. The amount of research focus in producing glycerol instead of ethanol suggest that this is, indeed, a basic alteration to consider, but depending on the type of vintage, the original microbiota, or the fermenting yeast strain, every case might require a specific approach, where this strategy should be combined with other of the described ones, or alternative ones yet to discover.
